# Transient Ectopic Overexpression of Agouti-Signalling Protein 1 (Asip1) Induces Pigment Anomalies in Flatfish

**DOI:** 10.1371/journal.pone.0048526

**Published:** 2012-12-10

**Authors:** Raúl Guillot, Rosa Maria Ceinos, Rosa Cal, Josep Rotllant, José Miguel Cerdá-Reverter

**Affiliations:** 1 Department of Fish Physiology and Biotechnology, Instituto de Acuicultura de Torre de la Sal, Consejo Superior de Investigaciones Científicas (IATS-CSIC), Castellón, Spain; 2 Aquatic Molecular Pathobiology Group, Instituto de Investigaciones Marinas, Consejo Superior de Investigaciones Científicas (IIM-CSIC), Vigo, Spain; 3 Instituto Español de Oceanografía de Vigo (IEO), Vigo, Spain; Universitat de Barcelona, Spain

## Abstract

While flatfish in the wild exhibit a pronounced countershading of the dorso-ventral pigment pattern, malpigmentation is commonly observed in reared animals. In fish, the dorso-ventral pigment polarity is achieved because a melanization inhibition factor (MIF) inhibits melanoblast differentiation and encourages iridophore proliferation in the ventrum. A previous work of our group suggested that asip1 is the uncharacterized MIF concerned. In order to further support this hypothesis, we have characterized asip1 mRNAs in both turbot and sole and used deduced peptide alignments to analyze the evolutionary history of the agouti-family of peptides. The putative asip precursors have the characteristics of a secreted protein, displaying a putative hydrophobic signal. Processing of the potential signal peptide produces mature proteins that include an N-terminal region, a basic central domain with a high proportion of lysine residues as well as a proline-rich region that immediately precedes the C-terminal poly-cysteine domain. The expression of asip1 mRNA in the ventral area was significantly higher than in the dorsal region. Similarly, the expression of asip1 within the unpigmented patches in the dorsal skin of pseudoalbino fish was higher than in the pigmented dorsal regions but similar to those levels observed in the ventral skin. In addition, the injection/electroporation of asip1 capped mRNA in both species induced long term dorsal skin paling, suggesting the inhibition of the melanogenic pathways. The data suggest that fish asip1 is involved in the dorsal-ventral pigment patterning in adult fish, where it induces the regulatory asymmetry involved in precursor differentiation into mature chromatophore. Adult dorsal pseudoalbinism seems to be the consequence of the expression of normal developmental pathways in an inaccurate position that results in unbalanced asip1 production levels. This, in turn, generates a ventral-like differentiation environment in dorsal regions.

## Introduction

In teleosts fish, pigment cells are commonly found in the dermis and can be divided into light-absorbing (melanophores, xantophores, erythrophores and cyanophores) and light-reflecting (leucophores and iridophores) chromatophores. Fish melanophores contain eumelanins (black-brown pigments), whereas xantophores and erytrophores synthesize carotenoids and/or pteridines that contribute to the reddish and yellowish components of the skin coloration. Iridophores are commonly localized in whitish and silvery areas of the skin, predominantly on the belly surface. They contain crystalline platelets composed of purines, mainly of guanine, which are responsible for the reflection of light [1]. Fish countershading is achieved by a patterned distribution of the pigment cells, with the light-absorbing and light-reflecting chromatophores mostly distributed in the dorsal and ventral areas, respectively [2], [3]. Although the pigment pattern is most evident in the adult animal, its cellular basis is established during embryogenesis [4]. Experimental data in fish and amphibian species suggest that this dorso-ventral pigment pattern is achieved because a putative diffusible melanization inhibition factor (MIF), locally produced by cells in the ventral skin, inhibits melanoblast differentiation and stimulates iridophore proliferation in the ventrum [2], [5], [6]. Our recent studies support agouti-signalling protein 1 (asip1) as the fish MIF [7]. *Asip1* encodes a 131 amino acid protein with structural characteristics of a secreted protein, which has a hydrophobic signal sequence and lacks a transmembrane domain. A highly basic domain with a high proportion of arginine and lysine residues forms the N-terminal region of the agouti protein. The latter region heads a proline-rich area that immediately precedes the cysteine-rich C-terminal domain. This cysteine domain resembles the conotoxins and plectoxins of snails and spiders, respectively [8].

In goldfish (*Carassius auratus*), *asip1* is expressed in the ventral skin but not in the dorsal skin. It inhibits melanocortin-induced melanin dispersion in melanocytes and selectively binds melanocortin receptor 1 (MC1R) [7]. This receptor shows high sensitivity to the melanocyte-stimulating hormone (α-MSH) and is profusely expressed within both dorsal and ventral skin [7], [9]. Interestingly, frameshift mutations introducing a premature stop codon in melanocortin MC1R or inactivating mutations in blind Mexican cave tetra (*Astyanax mexicanus*) are responsible for a decrease in the number of melanocytes and of the melanin content. This phenotype is recapitulated by MC1R knockdown in zebrafish [10]. Taken together, the data support that interaction between α-MSH/asip1 and MC1R is involved in the establishment of the dorsal-ventral pigment pattern, controlling chromatoblast survival, differentiation and/or proliferation as well as melanin synthesis.

Flatfish exhibit a pronounced countershading and are an excellent model to study the establishment of the dorso-ventral pigment pattern. These fish species undergo a metamorphosis from symmetrical free-swimming larvae to asymmetrical bottom-dwelling animals with both eyes on the same side. The dorsal-ocular side becomes dark pigmented whereas the ventral-blind side is white in color [11]. This pigment asymmetry appears in the adult stage and is hypothesized to depend upon the asymmetry of organizational environments that potentially regulate latent chromatophore precursor survival, proliferation and differentiation [11], [12]. Such regulatory asymmetry may be due to differences in the expression and distribution of secretory proteins involved in the precursor differentiation into mature chromatophore [13]. Accordingly, the common malpigmentation observed in reared flatfish, including pseudoalbinism (partial or total unpigmented ocular side) and hypermelanism (partial or total pigmented blind side), could be due to abnormalities in the asymmetry of the regulatory system [12], [14], [15]. The aim of this paper was to gain evidence supporting the view that *asip1* is able to generate a regulatory asymmetry that leads to dorsal-ventral pigment asymmetry. To this aim, we characterized sole (*Solea senegalensis*) and turbot (*Scophtalmus maximus*) *asip1* gene and analyzed tissue and developmental expression. We demonstrate that *asip1* is significantly more expressed in the ventral skin than in the dorsal skin. Moreover, when *asip1* is ectopically overexpressed in the ocular side it induces skin paling probably via inhibition of the melanogenic processes, whereas pseudoalbino animals exhibit increased *asip1* expression within the anomalous pigment areas.

## Results

### Cloning flatfish asip1 gene

Reverse transcription-polymerase chain reaction (RT-PCR) using degenerate primers designed by alignments of available fish *asip1* sequences; produced a partial cDNA fragment of 135 and 159 bp for sole and turbot, respectively. The putative translations exhibited high identity with the C-terminal cysteine domain of the published *asip1* sequences. To obtain the sequence of the complete peptide precursor RACE-PCR was performed in the 3′ and 5′ directions with specific primers. 3′ RACE generated unique bands of 422 and 499 bp for sole and turbot, respectively and provided information about the coding region of the exon 4 and the 3′ untranslated region. 5′ RACE experiments generated unique bands of 379 and 498 bp and provided information about the first *asip1* exons as well as the 5 ′untranslated region.

**Figure 1:**
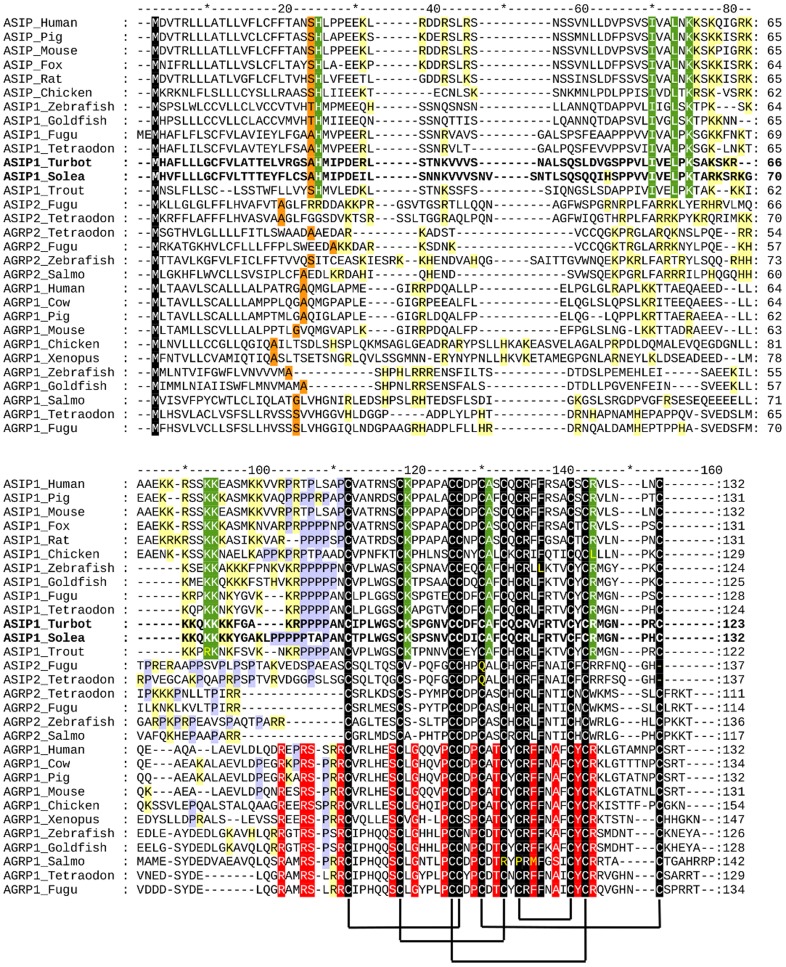
Alignment of agouti-signaling protein (asip) and agouti-related protein (agrp) amino acid sequences. Dashes were introduced to improve alignment. Orange boxes indicate the last residue of the predicted signal peptide. Black boxes show amino acid residues conserved in all sequences. Green boxes show residues only conserved in asip1 sequences. Red boxes indicate residues only conserved in agrp1 sequences. Yellow boxes indicate basic residues before cysteine domain. Blue boxes show residues of the short tail present in all agrp sequences. Purple boxes indicate putative glycosilation sites. Lines joining cysteine residues indicate putative disulfide bonds forming the cysteine domain. Arrow shows conserved motif for agrp post-transcriptional processing.

**Figure 2:**
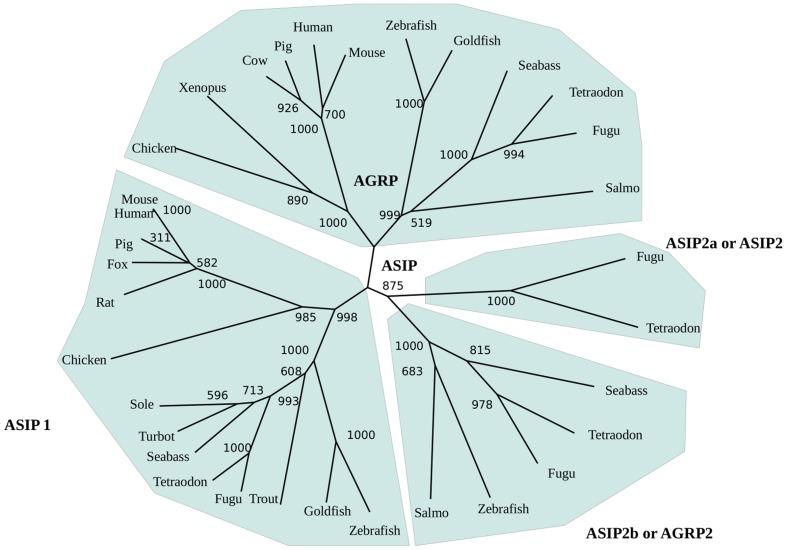
Phylogenetic tree of asip and agrp amino acid sequences built using CulstalX, which uses the Neighbor-Joining method on a matrix of distances. Numbers at branch nodes represent the confidence level of 1000 bootstrap replications. Phylogenetic analysis were done also by maximum likelihood using Seaview free software and no considerable differences were found.

The peptide precursors have the same organization as other species. The first 22 amino acids are estimated to constitute the signal peptide, which is followed by the 101 (turbot) or 110 (sole) amino acids of the mature peptide. One putative glycosylation sites were found within the highly basic N-terminal region of the sole but no glycosylation consensus sites were found in the turbot mature peptide. A proline-rich region and a poly-cysteine C-terminal domain followed the basic N-terminal region in both sequences. The poly-cysteine domain contains 10 cysteine residues with identical spatial pattern to that of agouti-like proteins, and similar to mammalian asip molecules it does not exhibit a short amino acid extension following the tenth cysteine residue (Fig. 1). Sole and turbot asip1 precursors were 73% identical. Flatfish amino-acid asip1 sequences are only 15–19% identical to asip2 of fish tetradontiform but they share 57–67% identical amino acids with asip1 precursor of the same species. The identity level of flatfish sequences with fish asip1 or asip2 was 18–20% and 15–19%, respectively. Phylogenetic analysis grouped flatfish asip1 sequences with the asip1 precursors of fish and tetrapod species. A different branch of the same cluster grouped asip2 and agrp2 sequences, whereas agrp precursors were grouped in a different cluster (Fig. 2).

### Temporal and spatial expression of *asip1*

**Figure 3:**
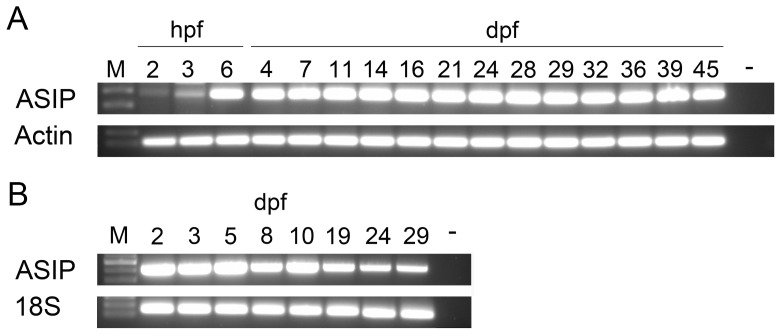
Expression of *asip 1* gene during early development. RT-PCR analysis of the temporal expression pattern of *a*sip1 in turbot (A) and sole (B). Hours post-fertilization, hpf; days post-fertilization, dpf.

The RT-PCR analysis (Fig. 3 A,B) showed that *asip1* transcripts existed maternally at a relatively low level, whereas zygotic expression persisted at relatively constant levels until the end of the sampling period (45 days post-fertilization, dpf) for turbot (Fig. 3A) and (29 dpf) sole (Fig. 3B).

**Figure 4:**
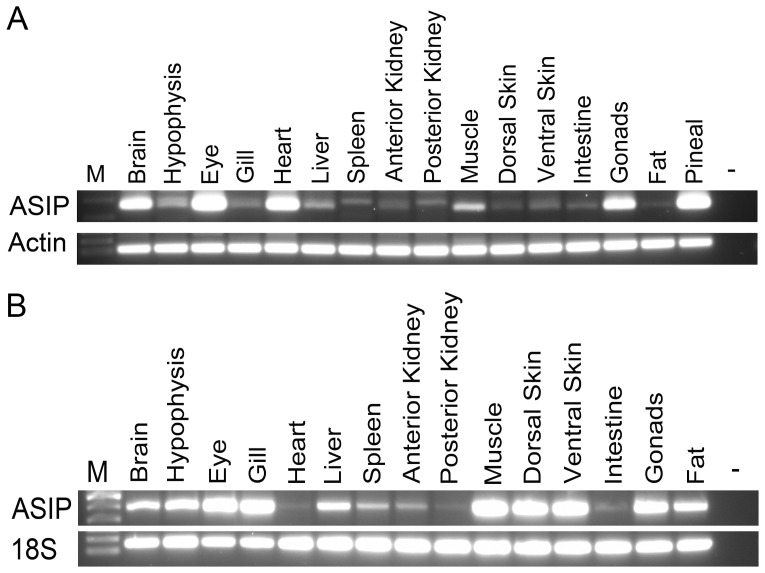
RT-PCR analysis of the tissue specific expression pattern of asip1. (A) Turbot and (B) sole.

At tissue level, *asip1* was highly expressed in the brain eye, heart, muscle, gonads and pineal organ of turbot. Very low expression levels were found in the hypophysis and liver. Residual levels were found in the remaining tested tissues including skin (Fig. 4A). Similar to turbot, sole *asip1* was expressed in the brain, hypophysis, eye, liver muscle and gonads but not in the heart. Additionally, high expression levels were detected in the gill, dorsal and ventral skin and adipose tissue (Fig. 4B).

### Spatially controlled expression of *asip1* gene

**Figure 5:**
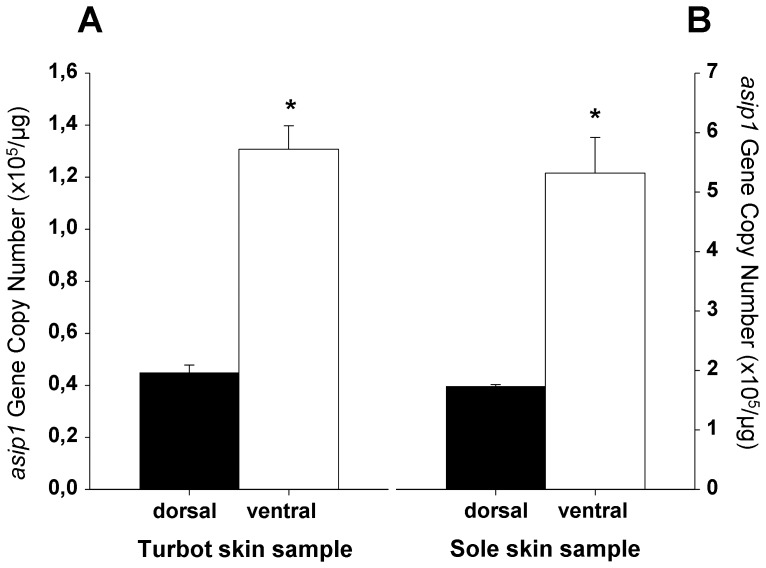
Analysis of differential dorsal-ventral *asip1* gene expression. Asip1 was differentially expressed in ventral non-pigmented skin or dorsal pigmented skin in turbot (A) and sole (B). *Asip1* gene expression was quantified by absolute qRT-PCR. The average *asip1* gene copy number per µg of primed cDNA was calculated from 5 individuals analyzed each time in triplicate. Data are expressed as mean ± SEM. Comparisons of numerical data were made by paired Student t-tests. *P<0.05.

To examine whether the expression of *asip1* gene is spatially regulated in turbot and sole skin, samples of dorsal and ventral skin were taken and *asip1* gene expression evaluated by absolute qRT-PCR. Consistent with the dorso-ventral expression pattern of *asip1* gene described in other fish species [7], the *asip1* transcripts were significantly more expressed in the ventral non-pigmented skin than in the dorsal pigmented skin of both fish species (Fig. 5A,B).

**Figure 6:**
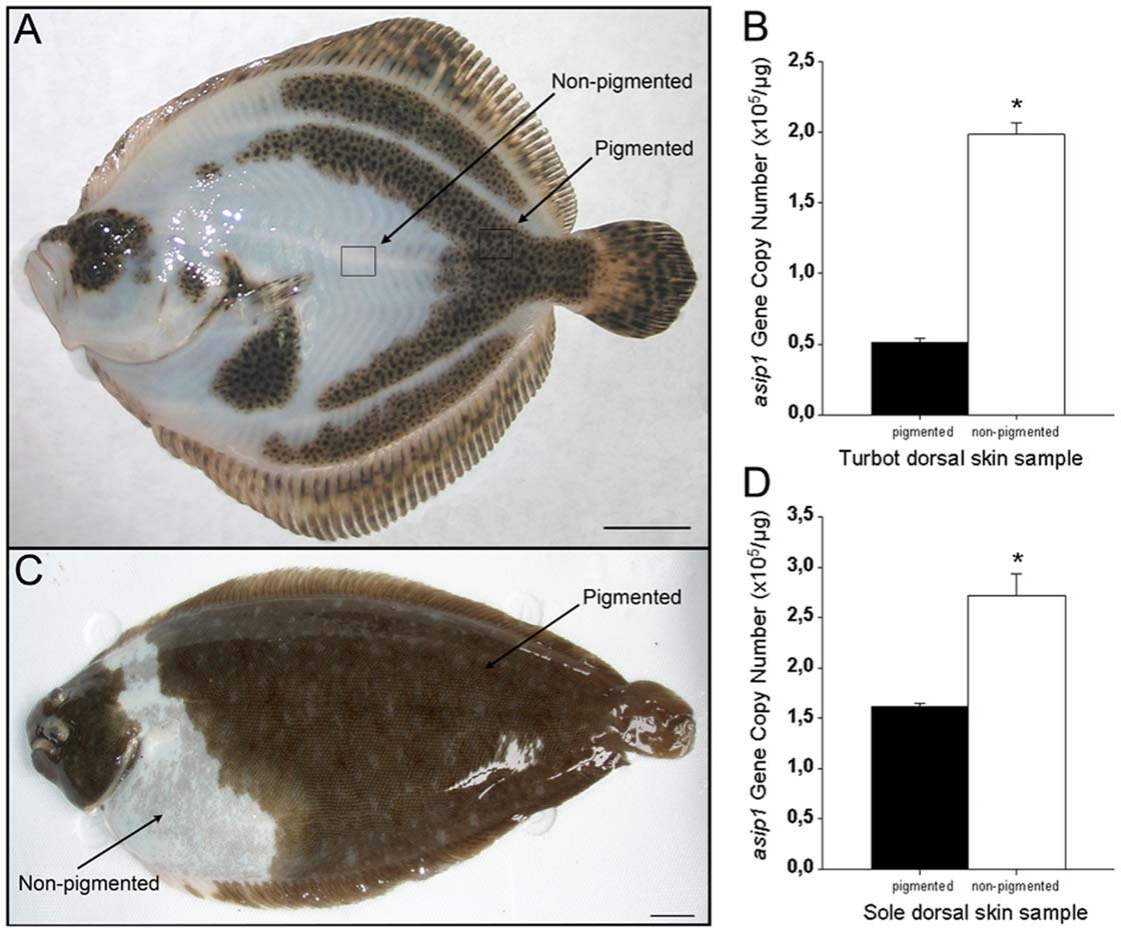
Analysis of *asip1* gene expression in pseudoalbino flatfish. Pseudo-albinism phenotype present in cultured turbot (A) and sole (C). *Asip1* was differentially expressed in non-pigmented white or pigmented brown dorsal skin areas in turbot (B) and sole (D). *Asip1* gene expression was quantified by absolute qRT-PCR. The average *asip1* gene copy number per µg of primed cDNA was calculated from 5 individuals analyzed each time in triplicate. Data are expressed as mean ± SEM. Comparisons of numerical data were made by paired Student t-tests. *P<0.05. Scale bars: 1 cm.

In pseudo-albino turbot (Fig. 6A) and sole (Fig. 6C), *asip1* gene expression was upregulated in dorsal non-pigmented regions compared with the dorsal pigmented regions in both turbot (Fig. 6B) and sole (Fig. 6D), suggesting a relationship of *asip1* gene expression levels and changes in skin pigmentation.

### Transient ectopic overexpression of *asip1* gene

**Figure 7:**
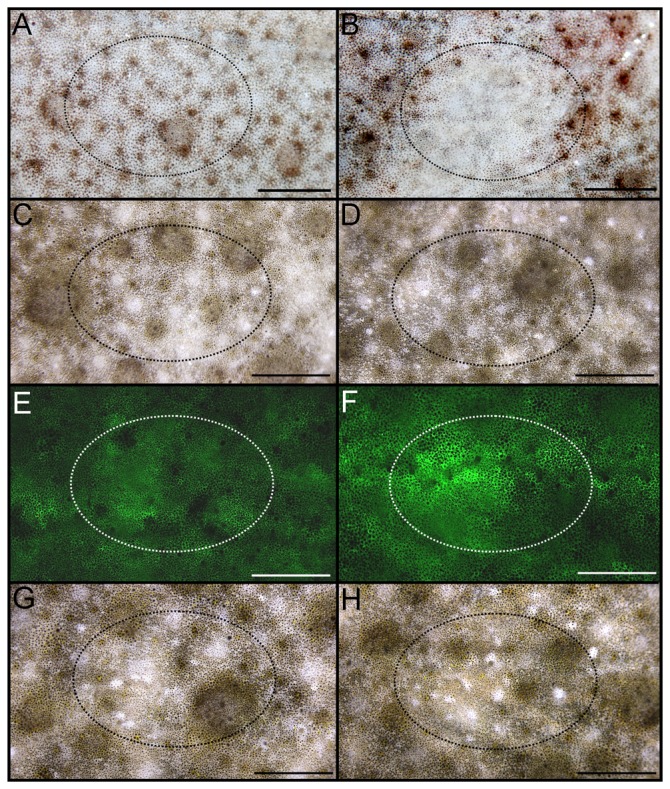
Views of dorsal skin of turbot injected and eletroporated *in vivo* to evaluate the effect of asip 1 overexpression on skin paling. Animals were injected with about 10 µg of capped sense (B) or antisense (D) mRNA per cm^2^ of dorsal skin and the effect was evaluated 4 days post injection/electroporation. Dorsal skin of control non-treated turbots are shown in A, C, E, and G. E and F show dorsal skin of control (E) and injected/electroporated animals with capped sense eGFP-mRNA (F) animals under fluorescent incident light w. G and H display animals shown in E and F under brilliant incident light. . Fluorescence was determined with a binocular Leica Stereoscope M165FC with digital camera (Leica Microsystem). Images were processed with Photoshop 7.0 (Adobe Systems) programs. Dorsal views, anterior to the right. Scale bars: 0.6 cm.

**Figure 8:**
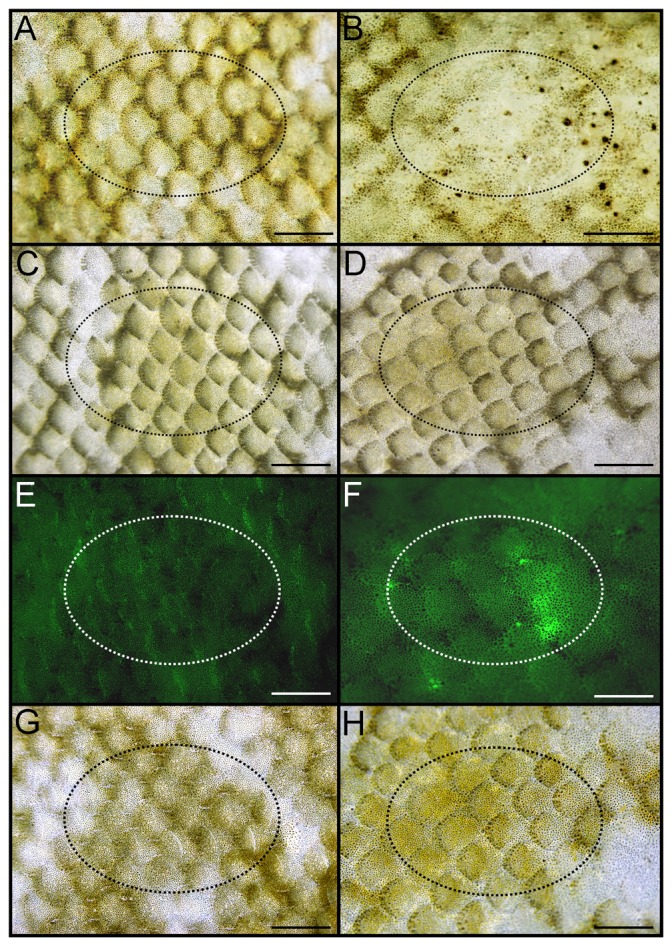
Views of dorsal skin of sole injected and eletroporated *in vivo* to evaluate the effect of asip 1 overexpression on skin paling. Animals were injected with about 10 µg of capped sense (B) or antisense (D) mRNA per cm^2^ of dorsal skin and the effect was evaluated 4 days post injection/electroporation. Dorsal skin of control non-treated turbots are shown in A, C, E, and G. E and F show dorsal skin of control (E) and injected/electroporated animals with capped sense eGFP-mRNA (F) animals under fluorescent incident light. G and H display animals shown in E and F under brilliant incident light. See Figure 7 for more details. Dorsal views, anterior to the right. Scale bars: 0.2 cm.

To investigate whether ectopic *asip1* expression could lead to pigment alteration on flatfish dorsal skin, we transiently overexpressed the *asip1* gene in turbot and sole dorsal skin area by *asip1-*capped mRNA injection and electroporation. The transient ectopic overexpression of *asip1* in the dorsal skin of turbot and sole induced a powerful paling of the skin 4 days after *asip1* gene overexpression (Fig. 7B; 8B). No skin pigmentation alteration was found in the antisense asip1-capped mRNA injected and electroporated fish (Fig. 7D; 8D) or *eGFP* (Fig. 7H; 8H) using brightfiled ilumination but increased fluorescence was evident in animals injected with sense *eGFP* (Fig. 7F; 8F). It means that sense *eGFP* injection and electroporation caused the expected effect without alteration of skin pigmentation.

**Figure 9:**
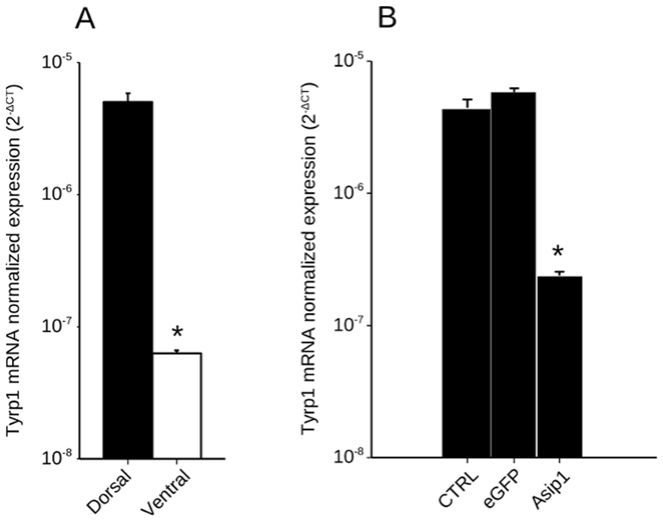
Normalized gene expression levels of tyrosinase-related protein 1 (Tyrp1) in turbot skin. (A) Analysis of differential dorsal-ventral Tyrp1 gene expression in turbot. (B) Effect of the *in vivo* injection of capped mRNA on Tyrp1 expression. Shown are log10 transformed ΔCt values of Tyrp1 relative to β-actin. Data are the mean ± SD from four samples after triplicate PCR analysis. Comparisons of numerical data were made by paired Student t-tests. Asterisk indicate significant differences (P<0.05) between dorsal and ventral Tyrp1 expression (A) and between control non-treated (CTRL) and eGFP capped mRNA injected skin Tyrp1 expression (B).

To confirm the effects of asip1 injection on melanogenic synthesis pathways, we studied tyrosinase-like protein 1 (Tyrp1) expression in intact, eGFP- and sense capped mRNA asip1-injected turbot skin. As expected Tyrp1 expression levels were lower in the ventral skin when compared to dorsal skin (Fig. 9A). Similarly the injection of sense asip1-capped mRNA, but no eGFP mRNA, induced a severe decrease in the Tyrp1 expression levels (Fig. 9B).

## Discussion

In this paper, we characterize *asip1* mRNA sequences for sole and turbot. *Asip1* is expressed in the main pigment tissues, i.e. eye and skin, but also in the central nervous system, including the pineal complex of turbot. Transitory overexpression of *asip1* mRNA in the melanic-dorsal side induces skin paling in both studied species and reduces the expression of key enzymes of the melanogenic pathway in turbot. Quantitative experiments demonstrated that *asip1* mRNA is overexpressed in non-pigmented regions of the dorsal skin in pseudoalbino turbot compared with melanic regions. The results demonstrate *asip1* participation in fish melanophore physiology and suggest its involvement in the organization of the dorsal-ventral pigment pattern.

Flatfish asip1 peptides keep the same structure exhibited by all agouti family of peptides. The putative asip precursors have the characteristics of a secreted protein, displaying a putative hydrophobic signal. Processing of the potential signal peptide produces 101 and 110-amino acid mature proteins in turbot and sole, respectively, including an N-terminal region, a basic central domain with a high proportion of lysine residues as well as a proline-rich region that immediately precedes the C-terminal poly-cysteine domain. Sole asip1 exhibited one potential N-glycosylation site within the N-terminal region asip but no consensus glycosylation sites were found in the turbot sequence. In mice, glycosylation of asip is an important factor for protein functionality as disruption partially reduces peptide activity in transgenic mice [16]. Similar to mammalian species, the basic domain of the sole and turbot peptides exhibit 10 lysine (K) and 2 and 3 arginine (R) residues, respectively. The integrity of this basic domain is also key for the full activity of the asip protein [17], [18]. The N-terminal region of mouse agouti has been shown to down-regulate melanocortin receptor signaling in *Xenopus* melanophore [19] and is also thought to mediate low affinity interactions with the product of the mahogany locus, i.e atracttin [20]. Spacing of the 10 cysteines within the C-terminal poly-cysteine domain is totally conserved in asip1 orthologues and the flatfish sequences are not an exception. This cysteine pattern resembles that of the conotoxins and agatoxins, suggesting that agouti-like proteins adopt an inhibitory cysteine knot (ICK) fold [21]. Structural studies have demonstrated that five disulfide bridges between cysteine residues, C87–C102, C94–C108, C101–119, C105–129 and C110–C117 stabilize the human agrp molecule [21]–[24]. Interestingly, Asip2 proteins lack the 5^th^ and 10^th^ cysteine residues which form the last disulfide bridge of agouti-like molecules. How these structural differences affect the dimensional conformation and receptor binding is unknown but we do know that the C-terminal loop in asip1 is required for MC1R binding [25].

Studies on the evolutionary history of the agouti family of peptides are controversial. Tetrapod species have two different melanocortin antagonists, i.e. *asip* and *agrp*, but teleost fish have four endogenous antagonists,. *asip1*, *asip2*, *agrp1* and *agrp2*. Studies have suggested that *asip2* and *agrp2* are ohnologue genes of *asip*1 and *agrp*1, respectively, which are generated during teleost-specific genome duplication (TSGD) [26]. Recent synteny data support the view that the *agrp2* chromosomal region does not share a synteny relationship with the fish *agrp*1 or with the tetrapod *agrp*. The *agrp2* and *asip2* regions show conserved synteny with a region of human chromosome 8 that, in turns, shares paralogies with the *asip* region on chromosome 20. The model proposes that the *agrp*/*asip* precursor was duplicated twice during the two rounds of vertebrate genome duplication (R1, R2). *Agrp2* and *asip2* were missed in the tetrapod genome but *asip*2 was retained in the teleost genome. After TSGD, the additional copy of *agrp* gene was missed again from the teleost genome but both copies of the *asip*2 gene were retained. These copies are named *asip2* and *agrp2* [26] but the new model proposes naming them *asip2a* and *asip2b*, respectively [27]. Schiöth and collaborators rebuilt the phylogeny by introducing a sequence from elephant shark [28]. If *agrp* is used to root the tree, the results support Braasch and Postlehwait's hypothesis but if the tree is rooted by the ancient sequence, the *agrp*2 and *asip*2 clusters group with the *agrp* cluster, supporting the previous nomenclature [26]. Flatfish sequences were grouped with asip1 sequences, suggesting their orthology. The incorporation of flatfish asip1 sequences does not modify the phylogeny reported by Braasch and Postlehwait's [27].

Structural and/or functional data could discern between both hypotheses. Human agrp is processed after the motif Arg^79^-Glu^80^-Pro^81^-Arg^82^ to release the active peptide (agrp 83–132 [29]. Both arginine (R) residues are fully conserved in all agrp sequences but not in asip1, asip2 and agrp2 sequences, which suggests that, unlike argp1 but similar to asip-like peptides, agrp2 peptides are not processed. The N-terminal region of asip peptides is rich in basic residues, particularly lysine (K). Similar to asip peptides, agrp2 peptides also exhibit a high number of basic residues before the cysteine domain. Asip1 sequences also present a proline domain immediately prior to the C-terminal cysteine domain. This domain is not present in agrp1 peptides and is not clearly defined in agrp2 or asip2 peptides. Also noteworthy is the fact that all asip2 and agrp2 sequences exhibit 5 residues between the second and third cysteine residue of the C-terminal domain, whereas asip1 and agrp1 peptides exhibit 6 residues. Therefore, structural data seem to support Braasch and Postlehwait's hypothesis defending the asip2/agrp2 ohnology and, by extension, the new nomenclature, asip2a/asip2b, respectively. However, one intriguing item of structural evidence disproves this reflexion. The alignment of peptide sequences show that all agrp sequences exhibit a short tail after the last cysteine residue and agrp2 sequences are not an exception. This short tail does not seem to confer any binding property to the molecule since the last twelve amino acids of the human agrp peptide can be eliminated without affecting MC3R or MC4R binding [21]. Therefore, the function of this conserved short C-terminal extension in all agrp1 and agrp2 sequences remains unknown. It is known that agouti peptides can interact with other molecules other than melanocortin receptors [20] suggesting a still undiscovered intermolecular interaction mediated by segments outside the cysteine-rich domain.

From the functional point of view, in mammalian species, a*grp* is expressed mainly in the hypothalamus where it regulates the energy balance. *Asip* is produced by dermal papillae cells in which it governs the switch between production of eumelanin and pheomelanin [30]. In fish all three *agrp*, i.e. *agrp*1, *agrp2* and *asip1* are expressed in the brain and skin [7], [26], [31]. In the brain, *agrp1* is exclusively expressed within the lateral tuberal nucleus, the fish homologue of the arcuate nucleus [31], whereas *agrp2* is only expressed in the pineal complex of zebrafish [32]. Our results demonstrate that, similar to *agrp2*, *asip1* is expressed in the turbot pineal complex. Coincident expression in the pineal complex can be expected if both genes derive from a common ancestral pineal-expressed gene, once again supporting a relationship between *argp2* and *asip* genes. As in other fish species, *asip1* was also expressed in the brain of flatfish. Specific *asip1*-expressing brain areas or projections, as well as the asip function in the brain, are unknown. We have previously shown that both MC4R [33], [34] and MC1R [9] are expressed in the fish brain. In addition, goldfish asip1 can bind both receptors [7], as agrp1 does [9], [34] thus significantly increasing the complexity of the central melanocortin signaling in fish.

In our previous studies, we proposed that asip could be the uncharacterized MIF in fish. We suggested that the ventral expression of asip induces an inhibitory effect on melanophore differentiation and/or proliferation but stimulates iridophore differentiation and/or proliferation via MC1R antagonism. Accordingly, the absence of asip expression in the dorsal skin allows melanoblasts to differentiate and/or proliferate, leading to dark coloration in the dorsal region [7]. In both sole and turbot, the expression of *asip1* in ventral skin was higher than in melanic dorsal skin. These findings are not so striking as those reported in goldfish, in which *asip1* expression in the dorsal skin was essentially absent. A possible reason for this discrepancy in dorsal/ventral relative expression levels could be the pigmental structure of the ocular side of flatfish. This side is normally patterned with dark patches and spots, as well as white and colored spots with a high number of iridophores, of all which are morphological entities (reviewed in [15]). Therefore, it is possible that *asip1* expression might also contribute to the dorsal heterogeneous pigment pattern in flatfish. In contrast, dorsal skin in goldfish is un-patched and much more homogeneous in dark pigmentation. We are currently testing the possibility that *asip1* might contribute to the pigment patterning outside the dorsal and ventral regions by comparing *asip1* expression in the lateral white and dark stripes of zebrafish.

We further demonstrated that transient *asip1* overexpression, following the injection of homologous capped mRNA, can induce skin paling in the dorsal melanic side of flatfish, *in vivo*. This result supports our hypothesis defending the involvement of asip1 protein in the patterning of dorsal-ventral pigmentation in fish. Our experimental design cannot elucidate whether the observed paling in turbot and sole skin was induced by a transient melanosome reorganization, similar to that observed during short-term background adaption or physiological color change, by a decrease in melanin synthesis or by a reduction in melanophore number, similar to that observed after long-term background adaptation or morphological color changes [15], [35]. All three scenarios are possible and could concur concomitantly. Experiments using recombinant goldfish asip1 demonstrated that this protein can inhibit melanin dispersion stimulated by melanocyte-stimulating hormone (MSH) in the melanophores of medaka scales in a reversible way [7]. However, our results show that treatment-induced effects persist even after 4 days post-administration, suggesting the presence of morphological color changes. Asip 1 overexpression induced a significant reduction in the Tyrp1 expression to reach similar levels to those exhibited in the ventral skin. Tyrp1 promotes final steps of eumelanin synthesis supporting that asip 1 overexpression inhibits melanogenesis and/or melanophore differentiation. Accordingly, asip1 has been shown to inhibit MSH-induced *mitf* expression, melanogenic gene promoters including tyrosinase, Tyrp1 and Tyrp2, melanoblast differentiation into melanocytes and induce melanocyte de-differentiation in mammals [36]–[38].

Capped mRNA administration experiments suggest a role for *asip1* in the adult pigment pattern of flatfish. Similar to *Danio* species, flatfish melanophores can be divided into larval, early or embryonic and adult or metamorphic-type melanophores [39]. During larval stages, pigment cell latent precursors are symmetrically located mainly along the dorsal and ventral margins of the flank and migrate continuously from these regions to the lateral sides. After late metamorphic stages, these precursors differentiate into adult-type chromatophore on the lateral asymmetrical sides. Pigment asymmetry in flatfish seems to depend upon an asymmetric organizational environment that may regulate survival, proliferation, distribution and differentiation of latent precursors into adult-type pigment cells since an asymmetric body plan, including eye migration, precedes adult pigment pattern formation [13], [40]. Recent studies in zebrafish have demonstrated that proliferative pigment cell precursors are associated with the peripheral nerve and ganglia and migrate to the hypodermis during pigment pattern metamorphosis, when they differentiate into melanophores or iridophores [41]. These precursors seem to be bipotential and thus capable of differentiating into melanophores or iridophores, depending on the interplay between forkhead transcription factor, *foxd3*, and microphthalmia subtype a, *mitfa*. Nacre zebrafish, a mutant for *mitfa*, exhibit an increased number of ectopic iridophores [42], while the loss of *foxd3*, a *mitfa* repressor, resulted in fewer iridophores [43], [44]. We hypothesized that, after migration, these bipotent precursors reach different developmental environments patterned by *asip1* expression, which finally governs the differentiation into melanophores or iridophores. There is no information about whether asip1 affects *foxd3* activity but we anticipate that asip1 could stimulate the expression of this *mitf* repressor. This model would not be only true for the tandem melanophore/iridophore since xantic goldfish lack dermic melanophore but display striking differences in the dorsal-ventral expression of *asip1* mRNA [7]. Therefore, a more plausible scenario is that asip1 could induce iridophore differentiation from bipotential melanophore/iridophore precursors which subsequently inhibit the differentiation of any type of chromatophore.

Pigment anomalies are common in reared flatfish including albinism of the ocular side and hypermelanism of the blind side. We demonstrated that asip expression in the albino regions of the ocular side in pseudoalbino turbots is similar to that observed in the ventral region but significantly higher than that seen in the dark areas of the ocular side. This suggests that ectopic expression of asip 1 could be involved in flatfish pseudoalbinism. It has been reported that albino flatfish, including turbot, are able to feed more efficiently and grow faster than controls (reviewed in [12]). This phenotype is recurrent to that observed in agouti mice carrying the unusual allele *A* ^y^. The associated phenotype is characterized by yellow fur and the ubiquitous expression of agouti gene, resulting in hyperphagia, hyperinsulinemia, increased linear growth, increased propensity for developing tumors, premature infertility and maturity-onset obesity [45], [46]. This metabolic syndrome is mediated by antagonizing α-MSH signaling at central MC4R that arbitrates the negative effects of melanocortin peptides on the energy balance [47]. We have previously demonstrated that central melanocortin system is involved in the regulation of the energy balance in fish via MC4R [31], [33], [48] and that asip1 can antagonize MSH effects on the latter receptors [7]. However, we cannot discriminate whether the increased expression levels of asip in the anomalous dorsal pigmental regions are the consequence of the expression of a normal developmental pathway in an incorrect position as result of a patterning error. It means, albino areas expressing higher asip1 mRNA levels within the melanic ocular side are indeed a portion of wrong-patterned ventral skin in dorsal position or, in other words, dorsal skin following the ventral skin developmental pathway. Asip1 expression levels in ventral skin and albino areas of the dorsal skin of turbot were similar. In addition, preliminary experiments further demonstrated that injection of cappep Asip1 mRNA into the hypermelanic regions of the ventral skin of sole inhibited melanogenesis (unpublished data; Guillot R, Ceinos, R, Rotllant, J and Cerdá-Reverter, JM).

In summary, we have characterized asip1 mRNAs in both turbot and sole and used deduced peptide alignments to study the evolutionary history of the agouti-family of peptides. Structural and functional data suggest that agrp2 is more closely related to asip than agrp1 sequences. Data suggest that fish asip is involved in the dorsal-ventral pigment patterning in adult fish, where it induces the regulatory asymmetry involved in precursor differentiation into mature chromatophores. Adult dorsal pseudoalbinism seems to be the consequence of the expression of normal developmental pathways in an erroneous position, resulting in unbalanced asip production levels. These, in turn, generate a ventral-like differentiation environment in dorsal regions.

## Materials and Methods

### Experimental animals

Turbot (*Scophtalmus maximus*) and sole (*Solea senegalensis*) larvae reared under standard commercial conditions were provided by the Instituto Español de Oceanografia (IEO), Vigo, Spain. Control and pseudoalbino adult fish were also obtained from stocks of the IEO. Animals were anesthetized in 0.02% tricaine methasulfonate (MS-222) before any manipulation and sacrificed by rapid decapitation when required. All experiments were carried out in accordance with the principles published in the European animal directive (86/609/EEC) for the protection of experimental animals and approved by the Consejo Superior de Investigaciones Científicas (CSIC) ethics committee (project numbers AGL2010-22247-C03-01 to JMC-R and ALG2011-23581 to JR). Unless otherwise indicated, all reagents were purchased from Sigma (St Louis MO, USA).

### Molecular cloning of flatfish asip1 gene

Total RNA from ventral skin of sole and turbot was extracted with Tri-reargent and treated with RQ1-DNAse I (Promega). Subsequently, mRNA was isolated with polyATrack mRNA isolation system III (Promega) following the manufacturer's manual. Synthesis of cDNA was primed with random hexameres (Invitrogen) and was used as template for PCR reactions with degenerate primers. These primers were designed based on *asip1* sequences from different species. The primers used to amplify sole *asip1* were Multi_Agouti_Fw 5′ CCKCCTCCBSCBAACTGY 3′ and Multi_Agouti_Rv 5′ CCCATKCGRCARTARCASAC 3′. These primers did not work with turbot cDNA and new primers called Flatfish_Agouti were designed based on cloned fish *asip1* sequences. The latter primers had the sequence: Flatfish_Agouti_Fw 5′ CTCCTGCYAACTGCMYTYCCTT 3′ and Flatfish_Agouti_Rv: 5′ GGGTTGCCCATTCGRCAGWAACA 3′. Fragments of 135 bp and 159 bp for sole and turbot asip, respectively, were cloned into pGEM-T easy vector (Promega), sequenced and found to show a high similarity with fish *asip1* sequences. To resolve 5′ and 3′ ends of sole and turbot cDNAs, 5′ and 3′ rapid amplification of cDNA ends (RACE) were performed using the Smart-RACE PCR cDNA amplification system (Clontech) following the manufacturer's manual and specific primers designed according to the previously obtained sequences. Purified fragments were treated as above. To corroborate that 5′ and 3′ ends correspond to the same transcript, full *asip1* sequences were amplified using specific primers targeting the cDNA extremes. Full cDNAs were cloned and sequenced as before. The nucleotide sequences of turbot and sole asip1 have been deposited with EMBL Nucleotide Sequence Database under accession numbers HE598752 and HE598753, respectively.

### Tissue and larvae RNA isolation and RT-PCR

Total RNA was purified as before. Superscript II reverse transcriptase (Invitrogen) was used for cDNA synthesis by priming total RNA from brain, hypophysis, pineal, eye, gill, spleen, anterior and posterior kidney, heart, liver, muscle, dorsal skin, ventral skin, intestine, gonads and fat with random hexameres (Invitrogen). PCR amplification was carried out with the primers specific primers amplifying the full coding region. As internal control of the reverse transcription step, PCR for β-actin (turbot) or 18S (sole) cDNA amplification was carried out. The following primer sequences were used; for sole, 18S forward primer had the sequence 5′ GAATTGACGGAAGGGCACCACCAG 3′ and 18S-reverse primer had the sequence 5′ ACTAAGAACGGCCATGCACCACCAC 3′. Turbot β-actin primers were β-actin-forward 5′ TGAACCCCAAAGCCAACAGG 3′ and β-actin-reverse 5′CAGAGGCATACAGGGACAGCAC 3′. Similarly, RNA from embryos collected at 2, 3 and 6 hours post fertilization (hpf) and 4, 7, 11, 14, 16, 21, 24, 28, 29, 32, 36, 39 and 45 days post hatching (dph) for turbot and 2, 3, 5, 8, 10, 19, 24 and 29 dph for sole were extracted and cDNA was primed as before.

### Skin RNA isolation and absolute-quantitative real time PCR (qRT-PCR)

Dorsal and ventral skin samples from control and pseudoalbino turbot and sole were collected and total RNA was extracted as before. cDNA was synthesized from total RNA using superscript III (Invitrogen) according to manufacturer's recommendations.

Absolute quantification was used as a method to analyse the skin spatially specific expression of asip 1 genes. Sole and turbot asip1 cDNAs cloned into pGEM-T easy were used as standards. 10-fold serial dilutions of asip1 into pGEM-T, ranging from 1×10^5^ to 1×10^10^ copies/µL, were used to construct standard curves for both asip1 genes. The concentration of the dsDNA standards was measured using a fluorometer and the corresponding copy number was calculated following the Whetlan method [49]. Real time PCR quantification (qRT-PCR) was performed in 96-well optical plates in triplicate on an Applied Biosystems 7500 analyzer with Maxima SYBR Green qPCR master mix (Fermentas, Life Science). The total reaction volume was 25 µl with 12.5 µL of SYBR green, 0.5 µL of each primer, 9.5 µL of nuclease free water and 1 µL of cDNA template. After denaturation at 95°C for 10 min, 40 cycles of amplification were carried out with denaturation at 95°C for 15 s, annealing and elongation at 60°C for 1 min, followed by the melting curve analysis. The following primer sequences were used for qRT-PCR: for turbot *asip1* (5′primer/3′primer) 5′ CTGCGAACTGCATTCCCTTGT 3′ and 5′ TCAGCAGCGAGGGTTGCC 3′, for sole asip1 (5′primer/3′primer) 5′ GCACTCCCTTGTGGGGAAG 3′ and 5′ TCAGCAGTGTGGGTTGCC 3′. A standard curve was drawn by plotting the natural logarithms of the threshold cycle (C_T_) against the number of molecules, respectively. C_T_ was calculated under default settings for the real-time sequence detection software (Applied Biosystems). The equation drawn from the graph was used to calculate the precise number of specific *Asip1* cDNA molecules present per microgram of total primed cDNA, tested in the same reaction plate as the standard.

Turbot Tyrp1 gene expression was quantified by relative qRT-PCR. The level of β-actin mRNA was used as an internal reference for sample normalization. Two pairs of primers were used for amplification: Tyrp1 forward (5′ CCAGGTTCAGCAATGTATCC 3′) and Tyrp1 reverse (5′ GCCATTCGGCTTCATAAGAG 3′). Data were analyzed using the comparative cycle threshold method (CT method). Characteristics of the real time PCR (qRT-PCR) system was the same as used above.

### Capped mRNA synthesis, injection and electroporation

To generate capped mRNA, DNA fragments containing the Kozak sequence followed by entire ORF of turbot and sole *asip1* were generated by PCR. These DNA fragments were subcloned into the pCS2+ vector to generate the *asip1* overexpression plasmid DNAs (pCS2+*asip1*-Turbot and pCS2+*asip1*-Sole). The purified plasmids were dissolved in DNase free water and stored at −20°C until use. The pCS2+*asip1* plasmids were linearized by restriction with NotI and used for capped sense or antisense *asip1* mRNA synthesis using mMessage Machine kit (Ambion). Five and seven month-old turbot and sole, respectively were anesthetized and asip1 capped mRNA was injected into the dorsal skin area using a 1 ml Omnifix®-F syringes. Approximately 10 µg of capped-mRNA was injected per cm^2^ of dorsal skin. Immediately following injection, both dorsal and ventral halves were electroporated using a ECM 830 BTX electroporator (Harvard apparatus,Inc.). Electric pulses were applied by a pair of electrode disks (7 mm diameter) rigged on the tips of tweezers (pinsettes-Type electrode 524, BTX instrument). The following parameters were used: 5-msec pulses of 10 V with a 200 msec pause between pulses. Fish were then rapidly returned to their tanks for skin coloration analysis at 4 days post-electroporation (dpe). The mRNA for green fluorescent protein (eGFP), which was synthesized from pCS2+-eGFP, was injected-electroporated into the skin as control.

At 4 dpe, fluorescein uptake was monitored. Five fish were tested in all experiments.

### Data analysis and statistics

Specimens were observed and photographed under a Leica M165FC fluorescence stereoscope (Leica Microsystems, Germany) equipped Leica DFC 500 digital camera. Adobe Photoshop^™^ software was used to adjust contrast levels in all images.

Flatfish sequences were compiled with Generunner free software and compared with known *asip1* and agouti-related protein (*agrp*) sequences from the National Center for Biotechnology Information (NCBI) and ENSEMBL databases. Sequence alignments were performed using public domain ClustalX 2.1 and edited with GeneDoc software. Phylogenetic tree was derived using CulstalX and SeaView that uses the Neighbor-Joining method on a matrix of distances and maximum likelihood, respectively. The cleavage site for removal of the hydrophobic signal peptide was predicted using SignalP 3.0 (http://www.cbs.dtu.dk/services/SignalP/). Differences in gene expression were assayed by Student t-test and statistical significance was considered at *p*<0.05. Results are given as mean ± SEM.
